# Association of Malnutrition, as Defined by the PG-SGA, ESPEN 2015, and GLIM Criteria, With Complications in Esophageal Cancer Patients After Esophagectomy

**DOI:** 10.3389/fnut.2021.632546

**Published:** 2021-04-26

**Authors:** Liangyu Yin, Nian Cheng, Ping Chen, Mengyuan Zhang, Na Li, Xin Lin, Xiumei He, Yingjian Wang, Hongxia Xu, Wei Guo, Jie Liu

**Affiliations:** ^1^Department of Clinical Nutrition, Daping Hospital, Army Medical University (Third Military Medical University), Chongqing, China; ^2^Institute of Hepatopancreatobiliary Surgery, Southwest Hospital, Army Medical University (Third Military Medical University), Chongqing, China; ^3^Department of Thoracic Surgery, Daping Hospital, Army Medical University (Third Military Medical University), Chongqing, China

**Keywords:** GLIM, ESPEN, PG-SGA, esophageal cancer, complications, malnutrition, esophagectomy

## Abstract

**Background:** There are several approaches that can be used for the pre-treatment identification of malnutrition in oncology populations including the Patient-Generated Subjective Global Assessment (PG-SGA), the 2015 consensus statement by the European Society for Clinical Nutrition and Metabolism (ESPEN 2015) and the Global Leadership Initiative on Malnutrition (GLIM).

**Aims:** This study aimed to evaluate whether malnutrition, as defined by these three methods, can be used to predict complications in esophageal cancer (EC) patients after esophagectomy.

**Methods:** We performed a single center, observational cohort study that included 360 EC patients undergoing esophagectomy from December 2014 to November 2019 at Daping Hospital in China. The prevalence of malnutrition in the study population was prospectively defined using the PG-SGA (≥9 defined malnutrition), and retrospectively defined using the ESPEN 2015 and the GLIM. The prevalence of malnutrition and association with postoperative complications were compared in parallel for the three methods.

**Results:** The prevalence of malnutrition before surgery was 23.1% (83/360), 12.2% (44/360), and 33.3% (120/360) in the study population, as determined by the PG-SGA, the ESPEN 2015 and the GLIM, respectively. The PG-SGA and GLIM had higher diagnostic concordance (Kappa = 0.519, *P* < 0.001) compared to the ESPEN 2015 vs. GLIM (Kappa = 0.361, *P* < 0.001) and PG-SGA vs. ESPEN 2015 (Kappa = 0.297, *P* < 0.001). The overall incidence of postoperative complications for the study population was 58.1% (209/360). GLIM- and ESPEN 2015-defined malnutrition were both associated with the total number of postoperative complications in multivariable analyses. Moreover, GLIM-defined malnutrition exhibited the highest power to identify the incidence of complications among all independent predictors in a pooled analysis.

**Conclusion:** Among the PG-SGA, the ESPEN 2015 and the GLIM, the GLIM framework defines the highest prevalence rate of malnutrition and appears to be the optimal method for predicting postoperative complications in EC patients undergoing esophagectomy. These results support the importance of preoperatively identifying malnutrition using appropriate assessment tools, because it can facilitate the selection of management strategies that will optimize the clinical outcomes of EC patients.

## Introduction

Esophageal cancer (EC) remains the sixth most common cause of cancer-related death according to the latest global cancer statistics (1). China accounts for ~55% of the global EC-related deaths (2). However, due to the recent advances in both the prevention and treatment of the disease, the mortality of EC has been exhibiting a decreasing trend in China (2). Thus, strategies that can improve the postoperative complications (3), clinical outcomes (4) and quality of life (QOL) (5) for EC patients are garnering accumulating interest in the oncology community, with newer approaches utilizing inter- or multi-disciplinary cancer treatment (6, 7).

In oncology patients, malnutrition may be induced by the metabolic and physical effects of the cancer, or may be a side-effect of anti-cancer treatments (8, 9). The prevalence of malnutrition in EC patients has been estimated to be as high as 79% (10), which has a significant negative impact on the incidence of postoperative complications, tolerance to treatment, and survival (11, 12). Nutritional intervention has been proposed as a key component of the multi-disciplinary treatment of EC to improve the patients' outcomes (13–15). By precisely targeting the reversible elements involved in the development of malnutrition, it may be possible to prevent or reduce the incidence of complications (16). Thus, nutritional assessment has become an important part of the pretreatment assessment of patients and is necessary to assess the impact of nutritional intervention (17).

There are several potential approaches that can be used for the standardized assessment of malnutrition among EC patients, including the Patient-Generated Subjective Global Assessment (PG-SGA) (18), the 2015 consensus statement by the European Society for Clinical Nutrition and Metabolism (ESPEN 2015) (19) and the newly-proposed Global Leadership Initiative on Malnutrition (GLIM) criteria (20). To date, the PG-SGA is the only assessment tool that is specially designed to evaluate the nutritional status of oncology patients (21). The ESPEN 2015 and the GLIM criteria were proposed as guidelines to unify the diagnosis of malnutrition in patients with a wide spectrum of diseases, including cancer (22, 23). However, the prevalence of malnutrition as determined by the three methods and the relationship with the incidence rates of postoperative complications among EC patients remain largely unknown. In the present study, our main purpose was to evaluate whether malnutrition, as defined by the three methods, can be used to predict complications in EC patients after esophagectomy. The secondary objective was to describe the differences in the prevalence of malnutrition in EC patients, as defined by the three methods.

## Methods

### Study Design and Participants

This was a single center, observational cohort study. This study included the consecutive patients with esophageal cancer diagnosed from December 2014 to November 2019 at Daping Hospital, Army Medical University, China. The inclusion criteria were: adult patients (≥18 years old) who were treatment-naive and diagnosed with primary EC using imaging technology, cytology or histology; scheduled plan to receive radical esophagectomy; all data available in the medical records; written consent provided. The exclusion criteria were: patients who were confirmed to have another cancer type by postoperative pathology; metastatic EC; those who underwent preoperative chemoradiotherapy; refused to cooperate; whose surgery was canceled; and those with incomplete data required by the study. The study was approved by the Ethics Committee of Daping Hospital.

### Data Acquisition

Within the first 24 h of admission, the following information was acquired for all participants by a trained dietitian: a standard nutritional interview to record the demographic characteristics and recent nutritional information of the patient, including the nutritional risk screening 2002 (NRS2002) score (24), the Karnofsky Performance Status (KPS) score (25) and the Patient-Generated Subjective Global Assessment (PG-SGA) score (21). A PG-SGA ≥9 was defined to indicate malnutrition in the present study. Smoking was defined as active tobacco smoking. Alcohol drinkers were defined as those with regular alcohol consumption (at least once a week or more regular) during the past 1 year.

For the anthropometric measurements, body weight and height were measured in light indoor clothing without shoes, to the nearest 0.1 kg and 0.1 cm, respectively. The body mass index (BMI) was calculated as weight (kg)/height (m)^2^. The mid-arm circumference (MAC) and calf circumference (CC) were measured using a flexible and non-elastic tape. The triceps skinfold (TSF) thickness was measured using an adipometer (PZJ-01, Jiangsu, China). The mid-arm muscle circumference (MAMC) was calculated based on the measured values the MAC and TSF (formula: MAC-3.14 × TSF). The hand grip strength (HGS) was measured by an electronic hand grip dynamometer (CAMRY, Model EH101, Guangdong, China). Patients were asked to stand comfortably, then to perform three maximal isometric contractions 1 min apart using their non-dominant hand, and the highest value was recorded.

Laboratory test results were obtained in our hospital's clinical laboratory using fasting blood. All disease- and treatment-related information during hospitalization, such as the tumor stage, histological type, laboratory values and short-term outcome, were obtained from medical records. Postoperative complications in the present study were categorized using the ECCG consensus (26) and graded using the Clavien-Dindo classification (CDC) system (27). Follow-up was performed at three and 6 months to acquire the survival status after discharge.

Parenteral nutritional (PN) was defined as nutrients administered intravenously and consisting of a combination of amino acids, carbohydrates, and fats, or any amino acids infusion, or any lipid emulsion infusion. Enteral nutrition (EN) was defined as oral nutrient supplementation or tube feeding providing at least 10 kcal/kg/d, according to the guideline from the European Society of Parenteral and Enteral Nutrition (28). Overall, a minimum of 3d of PN or EN was required to define patients as having received nutritional support. EN and PN were administered simultaneously in some patients.

Hospitalization costs were defined as the sum of all cancer-related medical expenditures during the patient hospitalization, such as surgery and nutritional support. Emergency treatment meant that rescue was carried out during the hospitalization. Normal discharge referred to complete oral feeding at discharge, with no need for tube feeding, good wound healing and no need for dressing changes. Discharge with tube nutrition referred to the need for continued tube feeding when the patient was discharged from the hospital. The need for regular dressing changes after discharge meant that the wound hadn't healed by the time of discharge.

### PG-SGA

The PG-SGA consists of two sections, the first includes questions about recent weight loss, food intake, symptoms that could interfere with food intake and the physical activity level of the patients. Weight loss referred to an unintentional loss of body weight during the 1 and 6 months before admission, and the percent weight change was calculated as (current-previous weight/previous weight) ×100%. Where possible, the 1-month weight change was used for the analysis; otherwise, it was imputed from the 6-month weight change, with minimal impact on the interpretation of the results. For the second section, information was collected about the disease and the patient's metabolic needs based on the tumor characteristics and a physical examination of the patient. Each item of the PG-SGA has a separate score, and the individual items are added up to obtain the final score. A score ≥9 was defined as indicating malnutrition in this study due to the present cut-off score of 9 was appropriate for initiation of urgent nutrition intervention (29).

### ESPEN 2015

All patients were initially screened to assess whether they were at nutritional risk before using the ESPEN 2015. In the present study, the NRS2002 was used as the screening tool (24). Different from the PG-SGA, the ESPEN 2015 and the GLIM criteria, all of which were designed to assess/diagnose malnutrition, the NRS2002 was originally developed to identify patients who are likely to benefit from nutritional intervention. The NRS2002 is used as a routine method to assess patients' nutritional risk at our institution. For patients who have nutritional risk (NRS2002 ≥3 in the present study), there are two alternative measurements that can be used to diagnose malnutrition by the ESPEN 2015 criteria (19). Alternative one: BMI <18.5 kg/m^2^. Alternative two: Unintentional weight loss >10% of the total body weight loss indefinite of time or >5% over the last 3 months, combined with an age-related low BMI (<20 kg/m^2^ in <70 years or <22 kg/m^2^ in ≥ 70 years) or a fat-free mass index (FFMI) <17 kg/m^2^ in men and <15 kg/m^2^ in women. A body composition analysis was not available for all of the participants, thus the FFMI-based criterion was not evaluated in the present study.

### GLIM

The GLIM criteria and their use to diagnose malnutrition and grade its severity have been described previously (20). In brief, there are two components; phenotypic criteria and etiologic criteria. For patients who have nutritional risk (NRS2002 ≥3 in the present study), at least one phenotypic criterion and one etiologic criterion needed to be positive to establish a diagnosis of malnutrition. For the phenotypic criteria, weight loss was evaluated using the results of the nutritional interview. The BMI was classified according to the Asian standards noted in the GLIM criteria, and the reference value for a severely low BMI (BMI <17) were defined according to a previous study conducted in an Asian population (30). To evaluate whether there was a reduced muscle mass (RMM), the 15th percentile (p15) of the CC was calculated separately for each gender. A value < p15 (male, 30 cm; female, 29 cm) was defined as positive for RMM according to our previous study (31). The etiologic criteria were evaluated according to the GLIM definition (20). Since all patients were pathologically diagnosed with EC and were planning to receive surgical treatment, the disease burden was evaluated as positive for all patients.

### Concordance Between the GLIM, ESPEN 2015, and PG-SGA

The accuracy, sensitivity, specificity, and Kappa coefficients (Kappa) were calculated to evaluate the diagnostic agreement between the GLIM, ESPEN 2015, and PG-SGA. A Higher Kappa indicates a better agreement between the tested methods for identifying malnutrition.

### Statistical Analysis

Continuous variables were expressed as the means ± standard deviation and were compared using a *t*-test. The normality of continuous data was tested using the Shapiro-Wilk test and the variance equality was tested using Levene's test. Categorical variables were expressed as numbers (percentages) and were compared using the Chi-squared test. Multivariate logistic regression analysis was used to evaluate the association between malnutrition and postoperative complications. A Bayesian Information Criterion (BIC)-based stepwise method in both directions was used to screen for the optimal model. The relative importance of different variables in the logistic regression model was calculated using a random forest algorithm. Covariates and dependent variables in the logistic regression model were set as the input and response variables in the random forest model, respectively. Two independent metrics (the mean decrease accuracy and the mean decrease gini) were calculated, with higher values indicating a higher relative importance of the covariate in the model. All tests were two-sided and *P* < 0.05 was regarded as statistically significant. All analyses were performed using R software (version 3.6.3, http://www.rproject.org).

## Results

### Baseline Characteristics

The baseline characteristics of the study population are shown in Table 1. No significant differences were observed between the malnourished group and well-nourished group in terms of gender, smoking, alcohol consumption, family cancer history, histologic type, clinical stage, differentiation grade, cancer site, or type of surgery in any of the groups defined by any of the three methods (all *P* > 0.05). As expected, the KPS score was associated with the identification of malnutrition by the PG-SGA (*P* < 0.001), but was not associated with the classification based on the ESPEN 2015 or the GLIM. No difference was observed between the malnourished group and the well-nourished group for any of the three methods with regard to the use of nutritional support or pathway of enteral nutrition.

**Table 1:** Baseline characteristics of the study population stratified by the PG-SGA, GLIM, and ESPEN 2015.

	**PG-SGA (≥9 defined malnutrition)**			**GLIM**			**ESPEN 2015**		
**Characteristics**	**Malnourished (*n* = 83)**	**Well-nourished (*n* = 277)**	***P***	**Malnourished (*n* = 120)**	**Well-nourished (*n* = 240)**	***P***	**Malnourished (*n* = 44)**	**Well-nourished (*n* = 316)**	***P***
Age, mean ± SD	65.30 ± 7.83	63.71 ± 7.68	0.100	65.21 ± 8.22	63.51 ± 7.43	0.049	65.32 ± 8.04	63.90 ± 7.69	0.256
Gender, male (%)	65 (78.3)	226 (81.6)	0.613	94 (78.3)	197 (82.1)	0.478	32 (72.7)	259 (82.0)	0.210
Smoking, yes (%)	60 (72.3)	190 (68.6)	0.613	84 (70.0)	166 (69.2)	0.968	29 (65.9)	221 (69.9)	0.712
Alcohol drinking, yes (%)	39 (47.0)	113 (40.8)	0.381	52 (43.3)	100 (41.7)	0.850	17 (38.6)	135 (42.7)	0.725
Family cancer history, yes (%)	17 (20.5)	40 (14.4)	0.250	22 (18.3)	35 (14.6)	0.444	6 (13.6)	51 (16.1)	0.837
Histological type (%)			0.837			0.446			0.523
Adenocarcinoma	10 (12.0)	29 (10.5)		14 (11.7)	25 (10.4)		3 (6.8)	36 (11.4)	
Small cell	1 (1.2)	2 (0.7)		0 (0.0)	3 (1.2)		0 (0.0)	3 (0.9)	
Squamous cell	72 (86.7)	246 (88.8)		106 (88.3)	212 (88.3)		41 (93.2)	277 (87.7)	
Clinical stage (%)			0.725			0.258			0.229
I	9 (10.8)	41 (14.8)		14 (11.7)	36 (15.0)		9 (20.5)	41 (13.0)	
II	33 (39.8)	97 (35.0)		44 (36.7)	86 (35.8)		19 (43.2)	111 (35.1)	
III	33 (39.8)	116 (41.9)		47 (39.2)	102 (42.5)		14 (31.8)	135 (42.7)	
IV	8 (9.6)	23 (8.3)		15 (12.5)	16 (6.7)		2 (4.5)	29 (9.2)	
Differentiation grade (%)			0.271			0.658			0.193
Well	10 (12.0)	41 (14.8)		15 (12.5)	36 (15.0)		10 (22.7)	41 (13.0)	
Medium	41 (49.4)	155 (56.0)		64 (53.3)	132 (55.0)		23 (52.3)	173 (54.7)	
Poor	32 (38.6)	81 (29.2)		41 (34.2)	72 (30.0)		11 (25.0)	102 (32.3)	
Cancer site (%)			0.947			0.815			0.428
Gastric-esophageal	10 (12.0)	29 (10.5)		13 (10.8)	26 (10.8)		2 (4.5)	37 (11.7)	
Lower esophagus	21 (25.3)	69 (24.9)		30 (25.0)	60 (25.0)		10 (22.7)	80 (25.3)	
Medium esophagus	38 (45.8)	136 (49.1)		55 (45.8)	119 (49.6)		23 (52.3)	151 (47.8)	
Upper esophagus	14 (16.9)	43 (15.5)		22 (18.3)	35 (14.6)		9 (20.5)	48 (15.2)	
Surgery type (%)			0.137			0.770			0.829
Conventional thoracic surgery	3 (3.6)	2 (0.7)		1 (0.8)	4 (1.7)		1 (2.3)	4 (1.3)	
Endoscopic	76 (91.6)	259 (93.5)		113 (94.2)	222 (92.5)		41 (93.2)	294 (93.0)	
Surgical robot	4 (4.8)	16 (5.8)		6 (5.0)	14 (5.8)		2 (4.5)	18 (5.7)	
Surgery duration, min, mean ± SD	284.33 ± 78.80	290.97 ± 80.25	0.507	285.78 ± 75.66	291.27 ± 81.97	0.540	262.95 ± 79.46	293.13 ± 79.34	0.019
KPS score, mean ± SD	90.00 ± 7.81	94.69 ± 6.89	<0.001	92.58 ± 6.80	94.12 ± 7.60	0.061	92.27 ± 7.43	93.80 ± 7.36	0.199
Nutritional support after surgery (%)			0.724			0.538			0.553
PN+EN	77 (92.8)	251 (90.6)		112 (93.3)	216 (90.0)		42 (95.5)	286 (90.5)	
PN+ONS	2 (2.4)	12 (4.3)		3 (2.5)	11 (4.6)		1 (2.3)	13 (4.1)	
TPN	4 (4.8)	14 (5.1)		5 (4.2)	13 (5.4)		1 (2.3)	17 (5.4)	
EN pathway (%)			0.438			0.658			0.234
Jejunostomy	31 (37.3)	79 (28.5)		40 (33.3)	70 (29.2)		19 (43.2)	91 (28.8)	
Nasoduodenal	46 (55.4)	172 (62.1)		72 (60.0)	146 (60.8)		23 (52.3)	195 (61.7)	
No EN	4 (4.8)	14 (5.1)		5 (4.2)	13 (5.4)		1 (2.3)	17 (5.4)	
ONS	2 (2.4)	12 (4.3)		3 (2.5)	11 (4.6)		1 (2.3)	13 (4.1)	

*PG-SGA, the Patient-Generated Subjective Global Assessment; GLIM, the Global Leadership Initiative on Malnutrition; ESPEN 2015, the 2015 consensus statement by the European Society for Clinical Nutrition and Metabolism; SD, standard deviation; KPS, the Karnofsky Performance Status; PN, parenteral nutrition; EN, enteral nutrition; ONS, oral nutritional supplements; TPN, total parenteral nutrition*.

### Prevalence of Malnutrition and Diagnostic Concordance of the Three Methods

**Figure 1:**
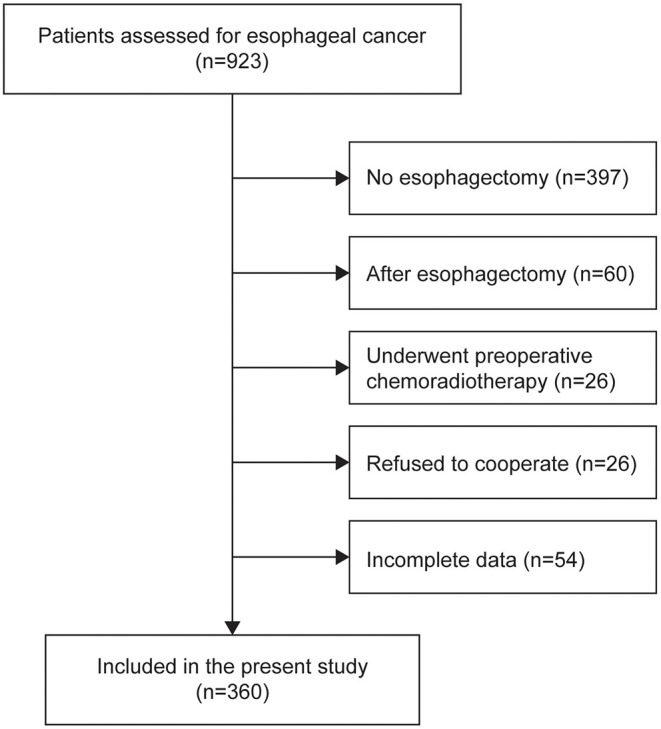
A flow chart of the patient inclusion.

**Figure 2:**
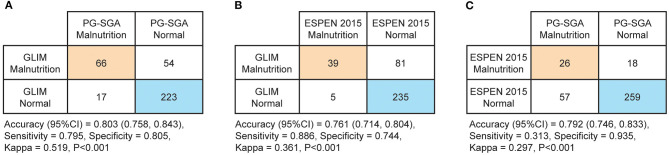
Diagnostic concordances between the Patient-Generated Subjective Global Assessment (PG-SGA, ≥9 defined malnutrition), the 2015 consensus statement by the European Society for Clinical Nutrition and Metabolism (ESPEN 2015) and the Global Leadership Initiative on Malnutrition (GLIM). **(A)** Diagnostic concordance between the GLIM and the PG-SGA **(B)** Diagnostic concordance between the GLIM and the ESPEN 2015 **(C)** Diagnostic concordance between the ESPEN 2015 and the PG-SGA.

In total, 360 patients were included in the present study (64.08 ± 7.74 years old; 80.8% male) (Figure 1). Malnutrition was diagnosed in 83 (23.1%), 44 (12.2%), and 120 (33.3%) of the patients using the PG-SGA, the ESPEN 2015 and the GLIM, respectively. When compared to the PG-SGA, the GLIM had an agreement (95%CI) of 0.803 (0.758, 0.843) for diagnosing malnutrition (sensitivity = 0.795, specificity = 0.805, Kappa = 0.519, *P* < 0.001, Figure 2A). Compared to the ESPEN 2015, the GLIM had an agreement (95%CI) of 0.761 (0.714, 0.804) for diagnosing malnutrition (sensitivity = 0.886, specificity = 0.744, Kappa = 0.361, *P* < 0.001, Figure 2B). And compared to the PG-SGA, the ESPEN 2015 had an agreement (95%CI) of 0.792 (0.746, 0.833) for diagnosing malnutrition (sensitivity = 0.313, specificity = 0.935, Kappa = 0.297, *P* < 0.001, Figure 2C).

### Nutritional Status

The associations between the nutritional information and the three methods are shown in Table 2. Of all the characteristics noted, the BMI, MAC and TSF were negatively associated with the presence of malnutrition diagnosed by all three methods, while weight loss within 6 months, weight loss beyond 6 months and nutritional risk were positively associated with the presence of malnutrition. The HGS was negatively associated with the ESPEN 2015-defined malnutrition, but such an association was not observed for the PG-SGA or the GLIM. The MAMC was negatively associated with the ESPEN 2015- and GLIM-defined malnutrition, but not with the PG-SGA. Reduced food intake within 1 month, reduced food intake at present and gastrointestinal condition were positively associated with the PG-SGA- and the GLIM-defined malnutrition, but not with malnutrition defined by the ESPEN 2015. No difference was observed between the malnourished group and the well-nourished group for any of the three methods in calf circumference.

**Table 2:** Nutritional characteristics of the study population stratified by the PG-SGA, GLIM, and ESPEN 2015.

	**PG-SGA (≥9 defined malnutrition)**			**GLIM**			**ESPEN 2015**		
**Characteristics**	**Malnourished (*n* = 83)**	**Well-nourished (*n* = 277)**	***P***	**Malnourished (*n* = 120)**	**Well-nourished (*n* = 240)**	***P***	**Malnourished (n=44)**	**Well-nourished (*n* = 316)**	***P***
BMI, kg/m^2^, mean ± SD	20.59 ± 2.78	22.90 ± 7.58	0.007	20.32 ± 2.56	23.39 ± 8.00	<0.001	18.08 ± 1.48	22.96 ± 7.08	<0.001
MAC, cm, mean ± SD	25.41 ± 4.27	26.62 ± 3.61	0.011	25.03 ± 3.75	27.00 ± 3.66	<0.001	22.99 ± 5.46	26.81 ± 3.26	<0.001
TSF, mm, mean ± SD	11.53 ± 5.26	13.88 ± 5.47	0.001	11.27 ± 4.77	14.38 ± 5.57	<0.001	10.52 ± 5.19	13.73 ± 5.44	<0.001
Handgrip strength, kg, mean ± SD	28.87 ± 24.78	28.64 ± 6.86	0.886	27.64 ± 20.95	29.22 ± 6.76	0.289	23.76 ± 7.15	29.38 ± 13.79	0.008
MAMC, cm, mean ± SD	21.79 ± 3.79	22.27 ± 3.29	0.263	21.49 ± 3.44	22.49 ± 3.35	0.008	19.69 ± 5.19	22.50 ± 2.93	<0.001
Calf circumference, cm, mean ± SD	31.94 ± 3.26	33.9a3 ± 16.01	0.262	33.24 ± 24.29	33.58 ± 2.63	0.829	36.54 ± 39.96	33.04 ± 2.93	0.124
Weight loss within 6 months, %, mean ± SD	5.54 ± 3.50	1.11 ± 1.91	<0.001	4.44 ± 3.73	0.98 ± 1.64	<0.001	5.20 ± 3.94	1.71 ± 2.60	<0.001
Weight loss beyond 6 months, %, mean ± SD	8.58 ± 10.21	2.04 ± 2.87	<0.001	7.05 ± 9.04	1.79 ± 2.60	<0.001	9.44 ± 13.66	2.73 ± 3.47	<0.001
Reduced food intake within 1 month, yes (%)	80 (96.4)	164 (59.2)	<0.001	104 (86.7)	140 (58.3)	<0.001	30 (68.2)	214 (67.7)	1.000
Reduced food intake now, yes (%)	51 (61.4)	102 (36.8)	<0.001	62 (51.7)	91 (37.9)	0.018	21 (47.7)	132 (41.8)	0.558
Gastrointestinal condition, yes (%)	81 (97.6)	205 (74.0)	<0.001	112 (93.3)	174 (72.5)	<0.001	35 (79.5)	251 (79.4)	1.000
NRS2002 ≥3, yes (%)	83 (100.0)	170 (61.4)	<0.001	120 (100.0)	133 (55.4)	<0.001	44 (100.0)	209 (66.1)	<0.001
Creatinine, μmol/L, mean ± SD	71.41 ± 19.06	70.52 ± 20.38	0.724	72.04 ± 21.56	70.06 ± 19.28	0.377	74.74 ± 36.92	70.16 ± 16.43	0.157
Albumin, g/L, mean ± SD	38.93 ± 9.55	43.48 ± 26.11	0.121	39.63 ± 6.84	43.83 ± 28.20	0.109	37.98 ± 8.44	43.05 ± 24.75	0.179
Prealbumin, mg/L, mean ± SD	221.34 ± 61.70	241.21 ± 83.04	0.044	221.45 ± 68.07	244.22 ± 83.03	0.010	231.90 ± 87.44	237.29 ± 77.88	0.672
C-reactive protein, mg/L, mean ± SD	4.17 ± 8.62	3.88 ± 5.10	0.713	3.92 ± 7.31	3.97 ± 5.38	0.940	3.72 ± 4.09	3.98 ± 6.31	0.787
Hemoglobin, g/L, mean ± SD	129.55 ± 17.58	135.14 ± 18.00	0.013	130.70 ± 18.58	135.43 ± 17.58	0.019	122.91 ± 16.53	135.38 ± 17.73	<0.001
NLR, mean ± SD	3.39 ± 7.60	2.93 ± 3.59	0.450	3.33 ± 6.47	2.89 ± 3.72	0.411	2.43 ± 1.35	3.12 ± 5.10	0.373

*PG-SGA, the Patient-Generated Subjective Global Assessment; GLIM, the Global Leadership Initiative on Malnutrition; ESPEN 2015, the 2015 consensus statement by the European Society for Clinical Nutrition and Metabolism; SD, standard deviation; BMI, body mass index; MAC, mid-arm circumference; TSF, triceps skinfold thickness; MAMC, mid-arm muscle circumference; NRS2002, Nutritional Risk Screening 2002; NLR, neutrophil lymphocyte ratio*.

### Laboratory Findings

The associations between the laboratory findings of the study population and the three methods are shown in Table 2. The blood levels of hemoglobin were negatively associated with malnutrition defined by all three methods. The prealbumin levels were negatively associated with the PG-SGA- and GLIM-defined malnutrition, but not with the ESPEN 2015-defined malnutrition. Other laboratory indices, including albumin, C-reactive protein, creatinine, and the NLR were not associated with malnutrition defined by any of the three methods.

### Univariate Analysis of the Patient Outcomes

The outcomes of patients during hospitalization, stratified by the presence of malnutrition as defined by the three methods, are shown in Table 3. The total incidence of complications for this study population was 58.1% (209/360). The main complications were pulmonary complications (41.4%), anastomotic fistulation (28.3%), infectious complications (11.7%), and atrial rhythm disorders (5.8%). The total incidence of complications, the incidence of pulmonary complications and the CDC severity grade of complications were positively associated with the presence of malnutrition as defined by all three methods. The incidence of infectious complications was positively associated with the GLIM-defined malnutrition, but not with malnutrition defined by the other methods. The incidence of anastomotic fistulation complications was positively associated with the ESPEN 2015-defined malnutrition, but not with the PG-SGA- or GLIM-diagnosed malnutrition. The incidence of atrial rhythm disorders was positively associated with the GLIM- and ESPEN 2015-defined malnutrition, but not with the PG-SGA-defined malnutrition. The incidence of other complications (as defined by the ECCG consensus) was positively associated with the PG-SGA and the GLIM-defined malnutrition, but not with the diagnosis based on the ESPEN 2015 criteria (Supplementary Table 1).

**Table 3:** In-hospital outcomes of the study population stratified by the PG-SGA, GLIM, and ESPEN 2015.

	**PG-SGA (≥9 defined malnutrition)**			**GLIM**			**ESPEN 2015**		
**Characteristics**	**Malnourished (*n* = 83)**	**Well-nourished (*n* = 277)**	***P***	**Malnourished (*n* = 120)**	**Well-nourished (*n* = 240)**	***P***	**Malnourished (*n* = 44)**	**Well-nourished (*n* = 316)**	***P***
ICU stay during hospitalization, yes (%)	7 (8.4)	20 (7.2)	0.896	11 (9.2)	16 (6.7)	0.524	2 (4.5)	25 (7.9)	0.625
Emergency treatment required during hospitalization, yes (%)	6 (7.2)	11 (4.0)	0.351	7 (5.8)	10 (4.2)	0.660	4 (9.1)	13 (4.1)	0.281
In-hospital death, yes (%)	3 (3.6)	5 (1.8)	0.578	3 (2.5)	5 (2.1)	1.000	2 (4.5)	6 (1.9)	0.569
Hospital stay total, days, mean ± SD	27.1 ± 14.6	29.3 ± 20.6	0.366	30.1 ± 17.9	28.1 ± 20.1	0.373	29.6 ± 17.6	28.7 ± 19.7	0.763
Discharge status (%)			0.105			0.048			0.226
Normal discharge	56 (67.5)	152 (54.9)		76 (63.3)	132 (55.0)		28 (63.6)	180 (57.0)	
Death	3 (3.6)	6 (2.2)		3 (2.5)	6 (2.5)		2 (4.5)	7 (2.2)	
Discharge with tube nutrition	10 (12.0)	62 (22.4)		14 (11.7)	58 (24.2)		4 (9.1)	68 (21.5)	
Dressing change regularly after discharge	14 (16.9)	57 (20.6)		27 (22.5)	44 (18.3)		10 (22.7)	61 (19.3)	
Revision surgery after esophagectomy, yes (%)	5 (6.0)	8 (2.9)	0.313	6 (5.0)	7 (2.9)	0.484	1 (2.3)	12 (3.8)	0.939
Hospitalization cost, dollars, mean ± SD	18,948.4 ± 716.2	19,992.8 ± 1372.6	0.483	19,843.6 ± 746.0	19,694.4 ± 1432.3	0.910	19,545.2 ± 671.4	19,843.6 ± 1313.0	0.877
Total postoperative complications, yes (%)	64 (77.1)	145 (52.3)	<0.001	102 (85.0)	107 (44.6)	<0.001	42 (95.5)	167 (52.8)	<0.001
Clavien-Dindo grade (%)			<0.001			<0.001			<0.001
I	1 (1.2)	18 (6.5)		1 (0.8)	18 (7.5)		0 (0.0)	19 (6.0)	
II	30 (36.1)	172 (62.1)		39 (32.5)	163 (67.9)		9 (20.5)	193 (61.1)	
III	39 (47.0)	65 (23.5)		61 (50.8)	43 (17.9)		29 (65.9)	75 (23.7)	
IV	11 (13.3)	17 (6.1)		17 (14.2)	11 (4.6)		5 (11.4)	23 (7.3)	
V	2 (2.4)	5 (1.8)		2 (1.7)	5 (2.1)		1 (2.3)	6 (1.9)	
Pulmonary, yes (%)	55 (66.3)	94 (33.9)	<0.001	85 (70.8)	64 (26.7)	<0.001	37 (84.1)	112 (35.4)	<0.001
Atrial dysrhythmia atrial requiring treatment, yes (%)	8 (9.6)	13 (4.7)	0.156	14 (11.7)	7 (2.9)	0.002	6 (13.6)	15 (4.7)	0.044
Leak from anastomosis, staple line or localized conduit necrosis, yes (%)	19 (22.9)	83 (30.0)	0.265	39 (32.5)	63 (26.2)	0.264	19 (43.2)	83 (26.3)	0.031
Infection, yes (%)	13 (15.7)	29 (10.5)	0.272	22 (18.3)	20 (8.3)	0.009	7 (15.9)	35 (11.1)	0.493

*PG-SGA, the Patient-Generated Subjective Global Assessment; GLIM, the Global Leadership Initiative on Malnutrition; ESPEN 2015, the 2015 consensus statement by the European Society for Clinical Nutrition and Metabolism; ICU, intensive care unit; SD, standard deviation*.

### Multivariate Analysis of the Patient Outcomes

**Figure 3:**
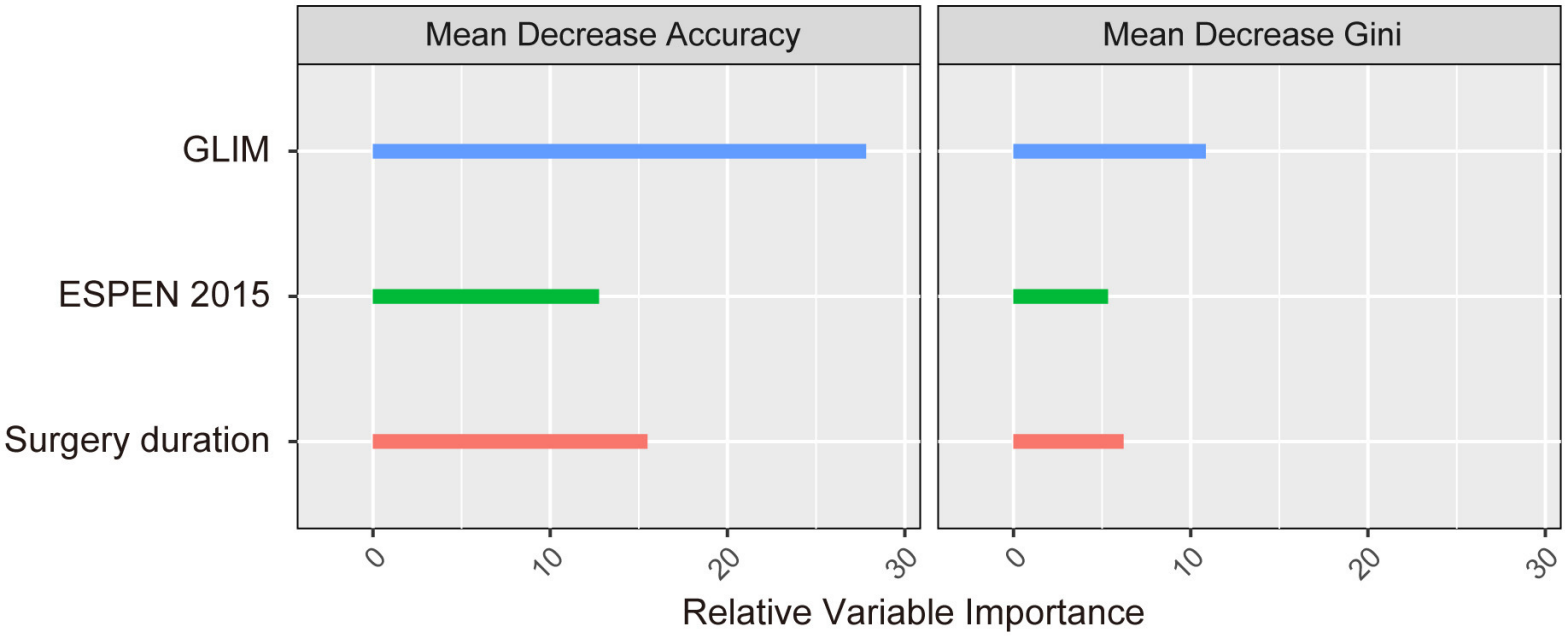
The relative importance of the independent predictors identified in the multivariable logistic regression model.

The association between malnutrition (as identified by the three methods) and the incidence of postoperative complications was subsequently analyzed using multivariable logistic regression analyses, and the results are shown in Table 4. We performed four independent analyses: the ability of the PG-SGA and other study characteristics to predict the total complications (the ESPEN 2015 and the GLIM were excluded, Model 1); the ability of the ESPEN 2015 and other study characteristics to predict the total complications (the PG-SGA and the GLIM were excluded, Model 2); the ability of the GLIM and other study characteristics to predict the total complications (the PG-SGA and the ESPEN 2015 were excluded, Model 3); the ability of all three methods and other study characteristics to predict the total complications (Model 4). The PG-SGA was not an independent predictor of complications, and was excluded during the BIC-based stepwise variable selection (Table 4, model 1). The ESPEN 2015-defined malnutrition was an independent risk factor for postoperative complications (OR = 15.84, 95%CI = 4.41–102.19, *P* < 0.001, Table 4, model 2). The results of model 3 showed that the GLIM-defined malnutrition was also an independent risk factor for postoperative complications (OR = 7.52, 95%CI = 4.30–13.77, *P* < 0.001). When all three methods were included, the GLIM (OR = 5.00, 95%CI = 2.79–9.35, *P* < 0.001) and the ESPEN 2015 (OR = 10.97, 95%CI = 2.89–72.86, *P* = 0.002) were both independent risk factors for postoperative complications, but the PG-SGA was not. An analysis of the relative importance of different variables in Model 4 showed that the GLIM contributed the most power (among the 3 covariates) to identify the incidence of postoperative complications, as indicated by both the mean decrease accuracy and the mean decrease gini metrics (Figure 3).

**Table 4:** Multivariable logistic regression analysis of the association between malnutrition identified by the PG-SGA, ESPEN 2015, GLIM, and total incidence of postoperative complications.

**Models**	**OR (95%CI)**	***P***
**Model 1, PG-SGA vs. total postoperative complications**		
Age, years	1.05 (1.02–1.08)	0.003
Weight loss within 1 month, %	1.28 (1.16–1.42)	<0.001
Mid-arm circumference, cm	0.90 (0.83–0.97)	0.005
Surgery duration, min	1.01 (1.00–1.01)	<0.001
**Model 2, ESPEN 2015 vs. total postoperative complications**		
Age, years	1.05 (1.02–1.08)	0.002
Weight loss within 1 month, %	1.22 (1.10–1.36)	<0.001
ESPEN 2015, malnourished vs. well-nourished	15.84(4.41–102.19)	<0.001
Surgery duration, min	1.01 (1.00–1.01)	<0.001
**Model 3, GLIM vs. total postoperative complications**		
Age, years	1.04 (1.01–1.07)	0.015
GLIM, malnourished vs. well-nourished	7.52(4.30–13.77)	<0.001
Surgery duration, min	1.01 (1.00–1.01)	0.001
**Model 4, all three methods vs. total postoperative complications**		
GLIM, malnourished vs. well-nourished	5.00 (2.79–9.35)	<0.001
ESPEN 2015, malnourished vs. well-nourished	10.97(2.89–72.86)	0.002
Surgery duration, min	1.01 (1.00–1.01)	<0.001

*PG-SGA, the Patient-Generated Subjective Global Assessment; GLIM, the Global Leadership Initiative on Malnutrition; ESPEN 2015, the 2015 consensus statement by the European Society for Clinical Nutrition and Metabolism*.

## Discussion

In this single center, observational cohort study, we evaluated the prevalence of malnutrition in EC patients undergoing esophagectomy using the PG-SGA, ESPEN 2015 and GLIM criteria, and compared the findings. Among the three methods, the GLIM criteria defined the highest baseline prevalence of malnutrition in the study population. Of interest, ESPEN 2015- and the GLIM-defined malnutrition were both independent risk factors for postoperative complications, but this association was not observed for the PG-SGA. The GLIM appears to be superior to other two methods to predict postoperative complications in EC patients undergoing esophagectomy.

The prevalence of malnutrition was 33.3, 12.2, and 23.1%, as determined by the GLIM, the ESPEN 2015 and the PG-SGA (≥9), respectively, in the study. A low concordance between the GLIM and the ESPEN 2015, the GLIM and the PG-SGA, and the ESPEN 2015 and PG-SGA was also observed in other studies (32–34). Possible explanations might include that: the PG-SGA is widely used as a nutritional assessment tool in cancer patients, while the GLIM and the ESPEN 2015 are diagnostic criteria for malnutrition of a variety of patients. A low BMI is considered to be one of the important criteria for diagnosing malnutrition in both the GLIM and the ESPEN 2015 criteria, whereas the BMI is not used in the PG-SGA. Furthermore, the PG-SGA is a subjective diagnostic tool developed by clinicians and patients, while the GLIM and the ESPEN 2015 are objective measurements which diagnose malnutrition using objective data and population-based cut-offs.

One possible reason for the low consistency between the GLIM and ESPEN standards may be that all of the standards considered in the ESPEN 2015 are included in the GLIM, while the GLIM also considers etiologic criteria. There is no consensus on the best way to measure and define a reduced muscle mass. As the recommended methods (dual-energy X-ray absorptiometry, bioelectrical impedance analysis, computed tomography, etc.) were not available for all of the patients in the present study, we used the 15th percentile (p15) of the CC, which was calculated separately for each gender, instead of the FFMI or SMI. While the ESPEN 2015 criteria require that weight loss is associated with a low BMI or low FFMI, we were unable to use the FFMI data in the present study since it was not available for all patients. This may have contributed to the differences in the diagnosis of malnutrition between the GLIM and the ESPEN 2015.

In our study, an analysis of the relative importance of different variables showed that the GLIM exhibited the greatest power to identify the incidence of postoperative complications among the three methods in model 4 (Table 4). The close relationship between malnutrition and negative clinical outcomes might be related to the evidence-based close relationship between the five individual criteria in the GLIM that are used in the new definition and malnutrition, while the ESPEN does not take into account inflammation, the disease burden, or reduced food intake/digestion due to gastrointestinal disorders and symptoms (35).

For non-metastatic EC, resection remains the cornerstone of treatment. Resection is often combined with neoadjuvant therapy. The incidence of complications associated with esophagectomy has previously been reported to range between 17 and 74% (36), which is directly related to surgical mortality, the cancer-related survival, length of hospital stay, readmission, hospitalization costs, and health-related quality of life (37–39). The risk of postoperative complications after esophagectomy is influenced by many factors, such as the type of surgery, tumor stage, preoperative nutritional status and so on (40). In the present study, complications after esophagectomy occurred in 58% of patients, similar to the results reported by Low et al. (41) and van der Werf et al. (42). The overall incidence of postoperative complications was higher than reported in other studies, possibly due to the adoption of the ECCG standard (26).

According to the GLIM criteria, there were significant differences in the rates of pulmonary complications, infectious complications, and atrial rhythm disorders between the malnourished group and the well-nourished group, but there was no difference in anastomotic fistula formation between the two groups. Pulmonary complications are the most common postoperative complications in patients with EC, and these are also one of the main causes of death in postoperative patients. In the current paper, the incidence of pulmonary complications was 41.4%, with pneumonia being the most common pulmonary complication (21.4%). Respiratory and swallowing muscles are affected by sarcopenia, and this reduced muscle capacity might result in pulmonary complications (43).

An anastomotic fistula is one of the most common and serious complications that can develop after resection of EC. There are many risk factors associated with postoperative anastomotic leakage, such as smoking, postoperative arrhythmia and other adverse cardiac events, the use of an Ivor-Lewis approach, advanced-stage cancer, lower preoperative albumin concentration, and so on (44). In our study, 149 patients were in clinical stage III or higher (41.4%), and 140 were over the age of 65 (38.9%). In addition, 70.3% of patients were at preoperative nutritional risk and 33.3% were malnourished according to the GLIM criteria, but preoperative nutrition intervention was not mandatory for these patients. These may explain why there was no significant difference in the incidence of anastomotic fistulation between the malnourished and well-nourished groups.

In conclusion, the GLIM framework defines the highest prevalence rate of malnutrition and appears to be the optimal method for predicting postoperative complications among the PG-SGA, the ESPEN 2015 and the GLIM methods in EC patients undergoing esophagectomy. These results emphasize the importance of the preoperative identification of malnutrition using an appropriate assessment tool in EC patients undergoing esophagectomy. Further research regarding the use of individualized nutritional intervention strategies in malnourished EC patients is needed to optimize their clinical outcomes after esophagectomy.

## Limitations

There are several potential limitations associated with this study. First, the study design was retrospective and we used medical records, which inherently involve missing data. Although the GLIM encourages the use of the criteria in prospective and retrospective cohort studies, as well as clinical trials, in order to validate its relevance for clinical practice, the risk of selection bias is not negligible. Second, the optimal cutoff values for reduced muscle mass remain a matter of debate. A body composition analysis was not available for all participants, so we used the 15th percentile (p15) of the CC, which was calculated separately for each gender, instead of the FFMI or SMI. The third limitation was that this study focused on short-term clinical outcomes, especially the rates of postoperative complications after esophagectomy. Long-term outcomes, such as patient survival, were not reported. We are currently conducting a prospective study to determine the prevalence of malnutrition in patients with EC and its association with clinical outcomes. Finally, due to the single center design and limited number of patients, future studies with a larger sample size and multicenter design are needed to replicate our results.

## Data Availability Statement

The datasets generated and/or analyzed during the current study are not publicly available to protect patient confidentiality but are available from the corresponding author on reasonable request.

## Ethics Statement

The studies involving human participants were reviewed and approved by The Ethics Committee of Daping Hospital. The patients/participants provided their written informed consent to participate in this study.

## Author Contributions

JL, HX, WG, and LY designed and conducted the research. JL, NC, PC, MZ, NL, XL, XH, and YW collected the data. LY, JL, and HX contributed to the interpretation of results and the manuscript preparation. LY contributed to the data analysis. All of the authors contributed to the review, editing, and approval of the final manuscript.

## Conflict of Interest

The authors declare that the research was conducted in the absence of any commercial or financial relationships that could be construed as a potential conflict of interest.
