# Supplementary material for: Association of Malnutrition, as Defined by the PG-SGA, ESPEN 2015, and GLIM Criteria, With Complications in Esophageal Cancer Patients After Esophagectomy
Source: Front Nutr. 2021 Apr 26;8:632546. doi: 10.3389/fnut.2021.632546 (PMC8107390; doi:10.3389/fnut.2021.632546)
Supplement: Supplementary file 1 [file Table_1.DOCX]

**Table S1. In-hospital outcomes of the study population stratified by the PG-SGA, GLIM and ESPEN 2015**

|  | PG-SGA (≥9 defined malnutrition) | | |  | GLIM | | |  | ESPEN 2015 | | |
| --- | --- | --- | --- | --- | --- | --- | --- | --- | --- | --- | --- |
| Characteristics | Malnourished  83 | Well-nourished  277 | *P* |  | Malnourished 120 | Well-nourished 240 | *P* |  | Malnourished 44 | Well-nourished 316 | *P* |
| ICU stay during hospitalization, yes (%) | 7 (8.4) | 20 (7.2) | 0.896 |  | 11 (9.2) | 16 (6.7) | 0.524 |  | 2 (4.5) | 25 (7.9) | 0.625 |
| Emergency treatment required during hospitalization, yes (%) | 6 (7.2) | 11 (4.0) | 0.351 |  | 7 (5.8) | 10 (4.2) | 0.660 |  | 4 (9.1) | 13 (4.1) | 0.281 |
| In-hospital death, yes (%) | 3 (3.6) | 5 (1.8) | 0.578 |  | 3 (2.5) | 5 (2.1) | 1.000 |  | 2 (4.5) | 6 (1.9) | 0.569 |
| Hospital stay after surgery, days, mean±SD | 21.01±13.76 | 22.53±18.83 | 0.496 |  | 23.49±17.32 | 21.52±18.01 | 0.323 |  | 22.95±16.72 | 22.07±17.95 | 0.758 |
| Hospital stay total, days, mean±SD | 27.10±14.59 | 29.29±20.61 | 0.366 |  | 30.07±17.89 | 28.14±20.10 | 0.373 |  | 29.61±17.60 | 28.67±19.65 | 0.763 |
| Discharge status (%) |  |  | 0.105 |  |  |  | 0.048 |  |  |  | 0.226 |
| Normal discharge | 56 (67.5) | 152 (54.9) |  |  | 76 (63.3) | 132 (55.0) |  |  | 28 (63.6) | 180 (57.0) |  |
| Death | 3 (3.6) | 6 (2.2) |  |  | 3 (2.5) | 6 (2.5) |  |  | 2 (4.5) | 7 (2.2) |  |
| Discharge with tube nutrition | 10 (12.0) | 62 (22.4) |  |  | 14 (11.7) | 58 (24.2) |  |  | 4 (9.1) | 68 (21.5) |  |
| Dressing change regularly after discharge | 14 (16.9) | 57 (20.6) |  |  | 27 (22.5) | 44 (18.3) |  |  | 10 (22.7) | 61 (19.3) |  |
| Revision surgery after esophagectomy, yes (%) | 5 (6.0) | 8 (2.9) | 0.313 |  | 6 (5.0) | 7 (2.9) | 0.484 |  | 1 (2.3) | 12 (3.8) | 0.939 |
| Hospitalization cost, dollars, mean±SD | 18,948.4± 716.2 | 19,992.8±  1372.6 | 0.483 |  | 19,843.6±  746.0 | 19,694.4±  1432.3 | 0.910 |  | 19,545.2±  671.4 | 19,843.6±  1313.0 | 0.877 |
| Total postoperative complications, yes (%) | 64 (77.1) | 145 (52.3) | <0.001 |  | 102 (85.0) | 107 (44.6) | <0.001 |  | 42 (95.5) | 167 (52.8) | <0.001 |
| Clavien-Dindo grade (%) |  |  | <0.001 |  |  |  | <0.001 |  |  |  | <0.001 |
| I | 1 (1.2) | 18 (6.5) |  |  | 1 (0.8) | 18 (7.5) |  |  | 0 (0.0) | 19 (6.0) |  |
| II | 30 (36.1) | 172 (62.1) |  |  | 39 (32.5) | 163 (67.9) |  |  | 9 (20.5) | 193 (61.1) |  |
| III | 39 (47.0) | 65 (23.5) |  |  | 61 (50.8) | 43 (17.9) |  |  | 29 (65.9) | 75 (23.7) |  |
| IV | 11 (13.3) | 17 (6.1) |  |  | 17 (14.2) | 11 (4.6) |  |  | 5 (11.4) | 23 (7.3) |  |
| V | 2 (2.4) | 5 (1.8) |  |  | 2 (1.7) | 5 (2.1) |  |  | 1 (2.3) | 6 (1.9) |  |
| **Pulmonary complication(s), yes (%)** | 55 (66.3) | 94 (33.9) | <0.001 |  | 85 (70.8) | 64 (26.7) | <0.001 |  | 37 (84.1) | 112 (35.4) | <0.001 |
| Pneumonia, yes (%) | 38 (45.8) | 39 (14.1) | <0.001 |  | 55 (45.8) | 22 (9.2) | <0.001 |  | 26 (59.1) | 51 (16.1) | <0.001 |
| Pleural effusion requiring additional drainage procedure, yes (%) | 20 (24.1) | 49 (17.7) | 0.254 |  | 35 (29.2) | 34 (14.2) | 0.001 |  | 15 (34.1) | 54 (17.1) | 0.013 |
| Pneumothorax requiring treatment, yes (%) | 10 (12.0) | 16 (5.8) | 0.090 |  | 17 (14.2) | 9 (3.8) | 0.001 |  | 8 (18.2) | 18 (5.7) | 0.007 |
| Atelectasis mucous plug requiring bronchoscopy, yes (%) | 3 (3.6) | 4 (1.4) | 0.422 |  | 5 (4.2) | 2 (0.8) | 0.079 |  | 4 (9.1) | 3 (0.9) | 0.002 |
| Respiratory failure requiring reintubation, yes (%) | 11 (13.3) | 21 (7.6) | 0.170 |  | 12 (10.0) | 20 (8.3) | 0.743 |  | 4 (9.1) | 28 (8.9) | 1.000 |
| Acute respiratory distress syndrome, yes (%) | 1 (1.2) | 9 (3.2) | 0.540 |  | 5 (4.2) | 5 (2.1) | 0.427 |  | 1 (2.3) | 9 (2.8) | 1.000 |
| Acute aspiration, yes (%) | 0 (0.0) | 2 (0.7) | 1.000 |  | 0 (0.0) | 2 (0.8) | 0.802 |  | 0 (0.0) | 2 (0.6) | 1.000 |
| Tracheobronchial injury, yes (%) | 0 (0.0) | 1 (0.4) | 1.000 |  | 0 (0.0) | 1 (0.4) | 1.000 |  | 0 (0.0) | 1 (0.3) | 1.000 |
| Chest tube maintenance for air leak for >10 d postoperatively, yes (%) | 1 (1.2) | 0 (0.0) | 0.522 |  | 1 (0.8) | 0 (0.0) | 0.723 |  | 1 (2.3) | 0 (0.0) | 0.248 |
| **Cardiac complication(s), yes (%)** | 9 (10.8) | 19 (6.9) | 0.339 |  | 14 (11.7) | 14 (5.8) | 0.082 |  | 6 (13.6) | 22 (7.0) | 0.212 |
| Cardiac arrest requiring CPR, yes (%) | 2 (2.4) | 3 (1.1) | 0.710 |  | 1 (0.8) | 4 (1.7) | 0.873 |  | 0 (0.0) | 5 (1.6) | 0.879 |
| Myocardial infarction, yes (%) | 0 (0.0) | 1 (0.4) | 1.000 |  | 0 (0.0) | 1 (0.4) | 1.000 |  | 0 (0.0) | 1 (0.3) | 1.000 |
| Atrial dysrhythmia requiring treatment, yes (%) | 8 (9.6) | 13 (4.7) | 0.156 |  | 14 (11.7) | 7 (2.9) | 0.002 |  | 6 (13.6) | 15 (4.7) | 0.044 |
| Ventricular dysrhythmia requiring treatment, yes (%) | 1 (1.2) | 2 (0.7) | 1.000 |  | 1 (0.8) | 2 (0.8) | 1.000 |  | 0 (0.0) | 3 (0.9) | 1.000 |
| Congestive heart failure requiring treatment, yes (%) | 0 (0.0) | 1 (0.4) | 1.000 |  | 0 (0.0) | 1 (0.4) | 1.000 |  | 0 (0.0) | 1 (0.3) | 1.000 |
| Pericarditis requiring treatment, no (%) | 83 (100.0) | 277 (100.0) | NA |  | 120 (100.0) | 240 (100.0) | NA |  | 44 (100.0) | 316 (100.0) | NA |
| **Gastrointestinal complication(s), yes (%)** | 22 (26.5) | 94 (33.9) | 0.256 |  | 47 (39.2) | 69 (28.7) | 0.061 |  | 22 (50.0) | 94 (29.7) | 0.012 |
| Esophagoenteric leak from anastomosis, staple line, or localized conduit necrosis, yes (%) | 19 (22.9) | 83 (30.0) | 0.265 |  | 39 (32.5) | 63 (26.2) | 0.264 |  | 19 (43.2) | 83 (26.3) | 0.031 |
| Conduit necrosis/failure, yes (%) | 1 (1.2) | 1 (0.4) | 0.948 |  | 1 (0.8) | 1 (0.4) | 1.000 |  | 0 (0.0) | 2 (0.6) | 1.000 |
| Ileus, deﬁned as small bowel dysfunction preventing or delaying enteral feeding, yes (%) | 3 (3.6) | 2 (0.7) | 0.150 |  | 5 (4.2) | 0 (0.0) | 0.007 |  | 1 (2.3) | 4 (1.3) | 1.000 |
| Small bowel obstruction, no (%) | 83 (100.0) | 277 (100.0) | NA |  | 120 (100.0) | 240 (100.0) | NA |  | 44 (100.0) | 316 (100.0) | NA |
| Feeding J-tube complication, yes (%) | 1 (1.2) | 7 (2.5) | 0.770 |  | 4 (3.3) | 4 (1.7) | 0.527 |  | 1 (2.3) | 7 (2.2) | 1.000 |
| Pyloromyotomy/pyloroplasty complication, no (%) | 83 (100.0) | 277 (100.0) | NA |  | 120 (100.0) | 240 (100.0) | NA |  | 44 (100.0) | 316 (100.0) | NA |
| Clostridium difﬁcile infection, no (%) | 83 (100.0) | 277 (100.0) | NA |  | 120 (100.0) | 240 (100.0) | NA |  | 44 (100.0) | 316 (100.0) | NA |
| Gastrointestinal bleeding requiring intervention or transfusion, yes (%) | 1 (1.2) | 7 (2.5) | 0.770 |  | 3 (2.5) | 5 (2.1) | 1.000 |  | 3 (6.8) | 5 (1.6) | 0.097 |
| Delayed conduit emptying requiring intervention or delaying discharge or requiring maintenance of NG drainage >7 d postoperatively, yes (%) | 0 (0.0) | 1 (0.4) | 1.000 |  | 0 (0.0) | 1 (0.4) | 1.000 |  | 0 (0.0) | 1 (0.3) | 1.000 |
| Pancreatitis, no (%) | 83 (100.0) | 277 (100.0) | NA |  | 120 (100.0) | 240 (100.0) | NA |  | 44 (100.0) | 316 (100.0) | NA |
| Liver dysfunction, yes (%) | 2 (2.4) | 8 (2.9) | 1.000 |  | 7 (5.8) | 3 (1.2) | 0.031 |  | 3 (6.8) | 7 (2.2) | 0.211 |
| **Urologic complication(s), yes (%)** | 3 (3.6) | 5 (1.8) | 0.578 |  | 5 (4.2) | 3 (1.2) | 0.164 |  | 1 (2.3) | 7 (2.2) | 1.000 |
| Acute renal insufﬁciency, yes (%) | 1 (1.2) | 2 (0.7) | 1.000 |  | 2 (1.7) | 1 (0.4) | 0.539 |  | 1 (2.3) | 2 (0.6) | 0.813 |
| Acute renal failure requiring dialysis, yes (%) | 2 (2.4) | 4 (1.4) | 0.909 |  | 4 (3.3) | 2 (0.8) | 0.190 |  | 0 (0.0) | 6 (1.9) | 0.769 |
| Urinary tract infection, no (%) | 83 (100.0) | 277 (100.0) | NA |  | 120 (100.0) | 240 (100.0) | NA |  | 44 (100.0) | 316 (100.0) | NA |
| Urinary retention requiring reinsertion of urinary catheter, delaying discharge, or discharge with urinary catheter, no (%) | 83 (100.0) | 277 (100.0) | NA |  | 120 (100.0) | 240 (100.0) | NA |  | 44 (100.0) | 316 (100.0) | NA |
| **Thromboembolic complication(s), yes (%)** | 2 (2.4) | 4 (1.4) | 0.909 |  | 3 (2.5) | 3 (1.2) | 0.662 |  | 2 (4.5) | 4 (1.3) | 0.335 |
| Deep venous thrombosis, yes (%) | 0 (0.0) | 3 (1.1) | 0.792 |  | 1 (0.8) | 2 (0.8) | 1.000 |  | 1 (2.3) | 2 (0.6) | 0.813 |
| Pulmonary embolus, yes (%) | 2 (2.4) | 3 (1.1) | 0.710 |  | 2 (1.7) | 3 (1.2) | 1.000 |  | 1 (2.3) | 4 (1.3) | 1.000 |
| Stroke, no (%) | 83 (100.0) | 277 (100.0) | NA |  | 120 (100.0) | 240 (100.0) | NA |  | 44 (100.0) | 316 (100.0) | NA |
| Peripheral thrombophlebitis, no (%) | 83 (100.0) | 277 (100.0) | NA |  | 120 (100.0) | 240 (100.0) | NA |  | 44 (100.0) | 316 (100.0) | NA |
| **Neurologic/psychiatric complication(s), yes (%)** | 2 (2.4) | 4 (1.4) | 0.909 |  | 4 (3.3) | 2 (0.8) | 0.190 |  | 0 (0.0) | 6 (1.9) | 0.769 |
| Recurrent nerve injury, no (%) | 83 (100.0) | 277 (100.0) | NA |  | 120 (100.0) | 240 (100.0) | NA |  | 44 (100.0) | 316 (100.0) | NA |
| Other neurologic injury, yes (%) | 2 (2.4) | 1 (0.4) | 0.266 |  | 3 (2.5) | 0 (0.0) | 0.065 |  | 0 (0.0) | 3 (0.9) | 1.000 |
| Acute delirium, yes (%) | 0 (0.0) | 2 (0.7) | 1.000 |  | 1 (0.8) | 1 (0.4) | 1.000 |  | 0 (0.0) | 2 (0.6) | 1.000 |
| Delirium tremens, yes (%) | 0 (0.0) | 1 (0.4) | 1.000 |  | 0 (0.0) | 1 (0.4) | 1.000 |  | 0 (0.0) | 1 (0.3) | 1.000 |
| **Infection, yes (%)** | 13 (15.7) | 29 (10.5) | 0.272 |  | 22 (18.3) | 20 (8.3) | 0.009 |  | 7 (15.9) | 35 (11.1) | 0.493 |
| Wound infection requiring opening the wound or antibiotics, yes (%) | 3 (3.6) | 7 (2.5) | 0.882 |  | 4 (3.3) | 6 (2.5) | 0.910 |  | 2 (4.5) | 8 (2.5) | 0.786 |
| Central IV line infection requiring removal or antibiotics, yes (%) | 0 (0.0) | 1 (0.4) | 1.000 |  | 1 (0.8) | 0 (0.0) | 0.723 |  | 0 (0.0) | 1 (0.3) | 1.000 |
| Intrathoracic/intra-abdominal abscess, yes (%) | 4 (4.8) | 15 (5.4) | 1.000 |  | 9 (7.5) | 10 (4.2) | 0.279 |  | 2 (4.5) | 17 (5.4) | 1.000 |
| Generalized sepsis, yes (%) | 1 (1.2) | 0 (0.0) | 0.522 |  | 1 (0.8) | 0 (0.0) | 0.723 |  | 0 (0.0) | 1 (0.3) | 1.000 |
| Other infection(s) requiring antibiotics, yes (%) | 5 (6.0) | 7 (2.5) | 0.227 |  | 7 (5.8) | 5 (2.1) | 0.119 |  | 3 (6.8) | 9 (2.8) | 0.354 |
| **Wound/diaphragm complication(s), yes (%)** | 2 (2.4) | 5 (1.8) | 1.000 |  | 3 (2.5) | 4 (1.7) | 0.893 |  | 0 (0.0) | 7 (2.2) | 0.679 |
| Thoracic wound dehiscence, yes (%) | 0 (0.0) | 2 (0.7) | 1.000 |  | 0 (0.0) | 2 (0.8) | 0.802 |  | 0 (0.0) | 2 (0.6) | 1.000 |
| Acute abdominal wall dehiscence/hernia, yes (%) | 2 (2.4) | 3 (1.1) | 0.710 |  | 3 (2.5) | 2 (0.8) | 0.426 |  | 0 (0.0) | 5 (1.6) | 0.879 |
| Acute diaphragmatic hernia, no (%) | 83 (100.0) | 277 (100.0) | NA |  | 120 (100.0) | 240 (100.0) | NA |  | 44 (100.0) | 316 (100.0) | NA |
| **Other complication(s), yes (%)** | 10 (12.0) | 14 (5.1) | 0.047 |  | 13 (10.8) | 11 (4.6) | 0.044 |  | 5 (11.4) | 19 (6.0) | 0.312 |
| Chyle leak, yes (%) | 4 (4.8) | 6 (2.2) | 0.363 |  | 5 (4.2) | 5 (2.1) | 0.427 |  | 1 (2.3) | 9 (2.8) | 1.000 |
| Reoperation for reasons other than bleeding, anastomotic leakage, or conduit necrosis, yes (%) | 2 (2.4) | 5 (1.8) | 1.000 |  | 4 (3.3) | 3 (1.2) | 0.345 |  | 1 (2.3) | 6 (1.9) | 1.000 |
| Multiple organ dysfunction syndrome, yes (%) | 4 (4.8) | 3 (1.1) | 0.087 |  | 4 (3.3) | 3 (1.2) | 0.345 |  | 3 (6.8) | 4 (1.3) | 0.055 |

PG-SGA, the Patient-Generated Subjective Global Assessment; GLIM, the Global Leadership Initiative on Malnutrition; ESPEN 2015, the 2015 consensus statement by the European Society for Clinical Nutrition and Metabolism; ICU, intensive care unit; SD, standard deviation; CPR, cardiopulmonary resuscitation; IV, intravenous; NG, nasogastric tube.
